# Emission-Programmed Femtosecond Bessel Beams for Fabricating Micro–Nano Hierarchical Structures

**DOI:** 10.3390/nano16040236

**Published:** 2026-02-12

**Authors:** Yu Lu, Lin Kai, Fei Yin, Qing Yang, Kaiduan Yue, Feng Chen

**Affiliations:** 1 State Key Laboratory for Manufacturing System Engineering and Shanxi Key Laboratory of Photonics Technology for Information, School of Electronic Science and Engineering, Xi’an Jiaotong University, Xi’an 710049, China; luyu90@xjtu.edu.cn (Y.L.); 3120105005@stu.xjtu.edu.cn (L.K.); 2 School of Instrument Science and Technology, Xi’an Jiaotong University, Xi’an 710049, China; yinfei@opt.ac.cn (F.Y.); yangqing@mail.xjtu.edu.cn (Q.Y.); yue@xjtu.edu.cn (K.Y.); 3 Xi’an Institute of Optics and Precision Mechanics (XIOPM), Chinese Academy of Sciences (CAS), Xi’an 710119, China

**Keywords:** micro–nano hierarchical structure, femtosecond laser manufacturing, Bessel beam

## Abstract

Ultrafast laser-induced micro–nano hierarchical structures show broad applicability in optoelectronics, functional surfaces, and biomedicine. However, precisely controlling their formation through light field manipulation remains a relatively unexplored area. This work demonstrates a rapid drilling strategy on silicon using an emission-programmed, high-repetition-rate femtosecond Bessel beam. This spatiotemporal modulation enables a unique manufacturing synergy that integrates subtractive drilling and thermo-fluidic redistribution by the central lobes with additive nanostructuring by the peripheral lobes, directly fabricating a micro–nano hierarchical structure comprising tapered micro-holes, elevated micropillars, and dense nanocoatings. Meanwhile, areal scanning enables programmable geometry control through line interval adjustment. This approach offers new insights into laser-matter interactions and facilitates applications in infrared photodetection or drag-reduction surfaces.

## 1. Introduction

Laser-generated micro–nano hierarchical structure holds significant potential across diverse fields such as energy, biomedicine, and engineering [1,2,3]. For instance, in black silicon, the micro-scale features enhance light-trapping through multiple reflections, where the nano-scale components broaden the absorption spectrum via subwavelength effects, collectively boosting photovoltaic efficiency [4,5,6]. In biomedical applications, the roles of micro–nano structures are distinct yet complementary. Microstructures function as templates for cell attachment and as lubricant reservoirs in anti-thrombogenic surfaces, while nanostructures are pivotal for stimulating cellular responses like osteoblast proliferation and osseointegration [7,8,9]. Similarly, in drag reduction, micro-scale riblet structures physically constrain near-surface fluid motion to reduce turbulence. Nano-textures may introduce superhydrophobicity, minimizing the contact area with water and thereby achieving substantial drag reduction [10,11,12]. Traditionally, the fabrication of diverse micro–nano hierarchical structures has primarily relied on adjusting laser parameters (e.g., fluence, repetition rate, and pulse width), scan trajectory, and the processing environment [13,14,15]. While effective in many processing scenarios, these approaches exhibit limitations under certain conditions. For example, nanostructures resulting from the in situ deposition of ablated material may exhibit limited adhesion, and achieving a firmly bonded interface typically requires multiple laser scanning passes [16,17]. Alternatively, the formation of well-ordered laser-induced periodic surface structures (LIPSS) requires moderate and stable periodic energy deposition. This condition is inherently difficult to maintain on complex or high-aspect-ratio microstructures; thus, regular LIPSS are largely restricted to relatively shallow features [18,19]. These constraints inherently restrict the architectural diversity and functional potential of manufacturable structures. While spatial light field modulation has recently emerged as a proven and powerful tool for generating various micro- or nano-scale features [20,21,22], its targeted application in the deliberate creation of micro–nano hierarchical structures has rarely been explored [23,24].

Recently, the Bessel beam stood as a highly successful form of spatially modulated light in ultrafast laser materials processing [25,26,27]. Capitalizing on its non-diffracting properties, namely, an extended focal depth and self-reconstructing ability, it enables single-step fabrication of micro–nanochannels with extreme aspect ratios [27,28]. While these characteristics are most readily exploited in transparent dielectrics like silica [29,30,31], sapphire [32], and polymers, including PMMA [33,34] and PET [35], the distinctive spatial energy distribution of Bessel beams nonetheless offers unique processing advantages when applied to opaque materials such as silicon and titanium [36,37,38,39]. In this work, using a rapid drilling-on-the-fly strategy with an emission-programmed, high-repetition-rate femtosecond Bessel beam, we demonstrate a unique micro–nano hierarchical structure that comprises tapered micro-holes, above-surface micropillars, and a dense coating of nanostructures. The systematic programming of fs pulse emission reveals that the underlying formation mechanism stems from a synergistic interaction between the central core and the side lobes of the Bessel beam, which integrates additive, transformative, and subtractive manufacturing processes into a single step. Specifically, the central lobes drive subtractive ablation and transformative material redistribution for microstructure shaping, while the energy deposited by the outer-side lobes facilitates the additive deposition and anchoring of the coating nanostructures. Finally, the resultant micro-nano hierarchical structure and its tunability under various processing parameters are investigated.

## 2. Materials and Methods

**Figure 1:**
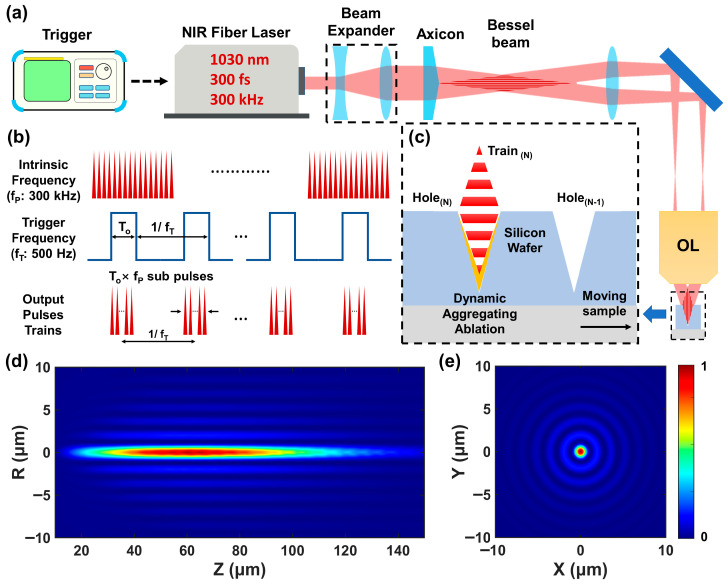
Rapid drilling strategy on a silicon wafer based on the high-repetitive-frequency femtosecond Bessel beam train by emission programming. (**a**) Experimental scheme. (**b**) Emission programming principle and strategy. (**c**) Rapid drilling-on-the-fly strategy. (**d**) Simulated transversal profile of the focused Bessel beam propagating in free space. (**e**) Simulated cross-section profile of the focused Bessel beam with Z = 40 μm in (**d**). OL: Objective Lens.

As depicted in Figure 1a, the rapid drilling strategy on silicon utilizes a temporally gated laser pulse train. Silicon was chosen for this study due to its well-characterized optical and thermal response, and its technological relevance in photovoltaics and photodetection ensures the practical significance of the approach. A near-infrared (NIR) fs fiber laser (FemtoYL-20, YSL Photonics, Wuhan, China; λ = 1030 nm; 300 fs) is operated at its intrinsic repetition rate of f_p_ (300 kHz in this work) and modulated by an external trigger signal. The pulse emission is enabled by a 5 V TTL and suppressed at 0 V, thus achieving programmable temporal sequences for processing. The Gaussian beam from the laser source was first expanded by a beam expander (comprising lenses of f = −100 mm and f = +150 mm focal lengths) and then spatially shaped into a Bessel beam using an Axicon (AX251-A, Thorlabs, Newton, NJ, USA; apex angle = 1.0°). This beam was subsequently demagnified and focused onto the surface of a silicon wafer through a 4f telescope system with a demagnification ratio of 40, comprising a long-focus lens (f = 160 mm) and an objective lens (DL-BMSOL-50x-0.65-PlnFlu, Dimension Labs, Shenzhen, China; 50×, NA = 0.65), for material processing. The timing of the trigger signal and the output pulses is illustrated in Figure 1b. For continuous processing, a square-wave trigger signal with a frequency of f_T_ = 500 Hz is used. The laser emits a sequence of pulse trains at a rate of f_T_, where each train contains N_p_ = T_o_ × f_p_ sub-pulses, with T_o_ denoting the high-level duration in each square wave. To investigate the formation mechanism of the micro–nano hierarchical structure, the number of sub-pulses per train and the total number of trains can be precisely adjusted by varying T_o_ or the number of square wave cycles, respectively. The rapid drilling process is illustrated in Figure 1c. Since silicon is opaque at the 1030 nm wavelength, single-shot drilling is unfeasible, which motivates the use of a dynamic rapid drilling-on-the-fly strategy that operates when continuously translating the sample. This approach leverages the cumulative effect of multiple sub-pulses within each pulse train: during the high-level duration T_o_, the sample displacement is intentionally limited to less than the effective drilling zone of the Bessel beam, establishing a quasi-static ablation regime that allows successive sub-pulses to collectively modify the material at an approximately fixed relative position. Each micro-hole is thus formed primarily by one dedicated pulse train. As the sample translates, the hole created by the (N−1)th train exits the primary interaction zone by the time the Nth train arrives, enabling the Nth train to initiate the same quasi-static process at a new, unprocessed location. These successive pulse trains facilitate the rapid fabrication of the micro-hole array. The transverse and cross-sectional intensity profiles of the Bessel beam are shown in Figure 1d,e, respectively. The transverse profile (Figure 1d) shows that the beam exhibits an extended depth of focus, maintaining its characteristic profile over a propagation length of about 120 μm. As illustrated in Figure 1e, the intensity profile on the silicon surface is dominated by an intense central lobe with a diameter of approximately 1.25 μm (bottom to peak), surrounded by concentric side-lobe rings. The majority of the beam energy is confined within the central lobe and the first few high-intensity side lobes. This unique intensity distribution underpins the spatially differentiated material interactions observed in this work.

## 3. Results

**Figure 2:**
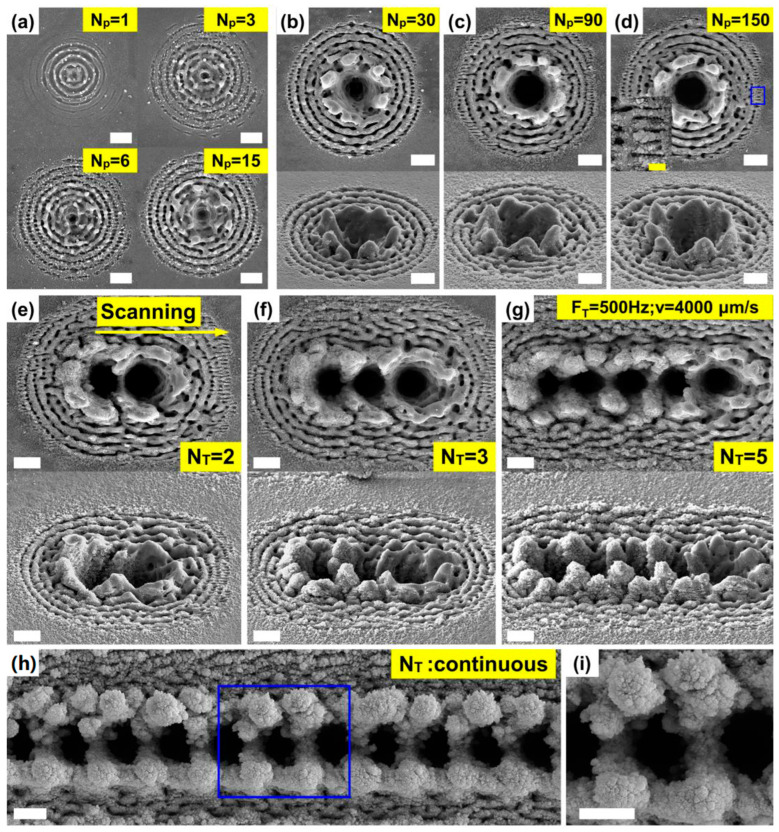
Quasi-reconstruction of the in-line rapid drilling processes based on the emission programming. (**a**–**d**) Quasi-reconstruction of the drilling processes within a single high-repetitive-frequency femtosecond Bessel beam train: (**a**) shows the SEM images of silicon morphologies with 1, 3, 6, and 15 sub-pulse aggregating in a single pulse train (N_p_); (**b**–**d**) show the SEM images with top (up) and oblique (down) view of silicon morphologies with (**b**) 30, (**c**) 90, and (**d**)150 sub-pulse aggregation. The inserted picture shows the magnified image of the LIPPS structures in (**d**). (**e**–**g**) Quasi-reconstruction of the in-line scanning processes by increasing the train numbers: Top SEM images indicate the top view, and down images indicate the oblique view; (**e**) two Bessel beam trains, N_T_ = 2; (**f**) three Bessel beam trains, N_T_ = 3; (**g**) five Bessel beam trains, N_T_ = 5. (**h**,**i**) In-line scanning results with continuous Bessel beam trains. Scale bar: 5 μm for the main figure; 1 μm for the inserted figure in (**d**). The moving speed of the sample was fixed at 4000 μm/s, and the incident sub-pulse energy was fixed at 15 μJ for all experiments depicted in this figure.

**Figure 3:**
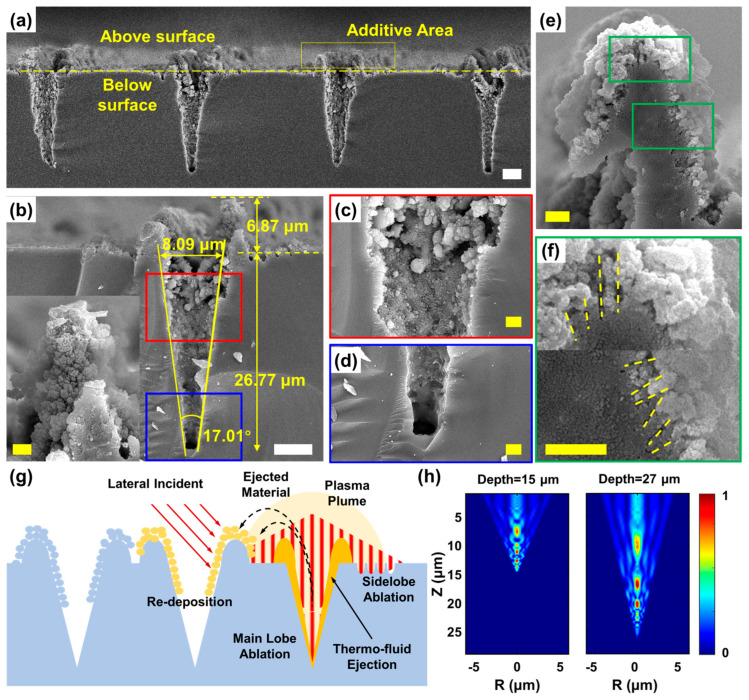
SEM image of the side view of the micro–nano hierarchical structure generated by in-line scanning with continuous Bessel beam trains. (**a**) Overview: SEM image of hierarchical structure. (**b**) High-magnification SEM image of a single hierarchical structure and its detailed morphologies; the inserted picture shows an entire micro-pillar (**c**) near the opening and (**d**) at the bottom. (**e**,**f**) Further magnified SEM images of the nanostructures deposited on the micro-pillars. (**g**) The underlying physical mechanism of the hierarchical structure generations: (**h**) Simulation of the e-field distribution inside a silicon V-groove incident with a focused Bessel beam. Scale bar: 5 μm for (**a**,**b**) (white ones); 1 μm for (**c**–**f**) and the inserted picture in (**b**) (yellow ones).

To decipher the cumulative dynamics of the rapid drilling process, we employed a strategy that quasi-reconstructs its progression by programming the fs pulse emission. The silicon surface was irradiated with an increasing number of sub-pulses (from 1 to 150) within a single train (N_p_), and the resultant morphologies were captured by SEM (Figure 2a–d). It is important to note that each micrograph captures the final morphology after the completion of a specific number of pulses, rather than an intermediate state during the process. Nevertheless, the progressive series of these morphologies effectively traces the key developmental stages across the multi-pulse ablation timeline, providing critical insights into the governing trends and transitional behaviors of the dynamic aggregating ablation mechanism. As shown in Figure 2a, a single sub-pulse leaves a distinct imprint of the Bessel beam’s intensity profile on the silicon surface, where the central main lobe and concentric side lobes are clearly resolved. As the number of sub-pulses increases to 15, this well-defined profile undergoes a marked transformation. In the central region, where the main lobe exhibits a peak fluence of 59 J/cm^2^, fluidic resolidification structures are formed, which disrupt the original annular patterns associated with the neighboring side lobes. Concurrently, the ablation structures in the outer-side lobe regions become increasingly distinct. Notably, within the areas corresponding to the sixth and seventh side lobes, where peak fluences range from 1.32 J/cm^2^ to 0.91 J/cm^2^, the emergence of laser-induced periodic surface structures (LIPSS) is observed. With a further increase in pulse count (Figure 2b–d), a prominent micro-hole forms in the central region, encompassing the area of the main lobe and several adjacent high-intensity side lobes. The central part of the hole appears progressively darker in the SEM images, suggesting a continuous increase in depth. The circumferential region of the hole is surrounded by resolidified ejecta, which, as evidenced by the oblique-view images, rise significantly above the original surface and grow in height with cumulative pulses. A magnified image of which is shown in the inserted picture of Figure 3d. As the number of pulses increases, the region of stable LIPSS generation extends further outward into the area corresponding to the eighth and nineth side lobes, where the peak fluences range from 0.57 J/cm^2^ to 0.35 J/cm^2^. Concurrently, the lower-order LIPSSs closer to the main lobe gradually transformed into groove structures. These observed trends in LIPSS evolution are consistent with the results reported in [40]. The surface morphologies resulting from in-line scanning with a limited number of Bessel beam trains are presented in Figure 2e–g. Given a constant sample translation speed of 4000 μm/s and a pulse train frequency, namely, the trigger frequency of f_T_ = 500 Hz, the center-to-center spacing between adjacent micro-holes is approximately 8 μm. When two pulse trains are applied (Figure 2e), the micro-hole drilled by the second train partially overlaps with the previous one. Furthermore, the ablation traces from the higher-order side lobes of the second train evidently extend over the area of the first hole. The microstructure surrounding the second hole resembles that formed by a single pulse train. However, the resolidified rim around the first hole exhibits a marked increase in nanofeature density. Since all samples underwent a 6 min ultrasonic cleaning prior to imaging, it can be confirmed that these nanostructures are firmly anchored to the underlying micro-features. Oblique-view observations further reveal that the rim around the first hole is notably taller than that of the second, likely a result of cumulative nanomaterial deposition. Similar progressive accumulation is observed as the number of pulse trains increases to three (Figure 2f) and five (Figure 2g). In these cases, the most recently formed hole consistently exhibits the fewest adherent nanostructures, whereas the earlier holes are decorated with significant nanoparticle accumulation. Moreover, with increasing cumulative pulses, the resolidified rims develop a distinct pattern: at the junctions where the rims of adjacent holes converge, the height becomes significantly greater, forming pillar-like structures that are particularly evident in the oblique views. When the pulse trains are delivered continuously, as shown in Figure 2h,i, the phenomena revealed in Figure 2e–g manifest in a continuous and highly reproducible manner, demonstrating the stability of this cumulative structuring process.

Cross-sectional side-view SEM images of the hierarchical structures are presented in Figure 3. As shown in Figure 3a, the cross-sections were revealed by cleaving the sample along multiple independent lines fabricated via in-line scanning. These images demonstrate high consistency across the structures, each featuring pillar-like protrusions above the original surface and underlying tapered micro-holes. Higher-magnification views (Figure 3b–d) provide quantitative insights: the surface opening of the tapered hole measures 8.09 μm, while the revealed depth reaches 26.77 μm. The geometry of the hole yields a core angle of 17.01°. Furthermore, the morphology reveals a distinct distribution of nanoscale features: abundant nanostructures coat the micropillars and the upper sections of the micro-holes, while the bottom of the tapered micro-hole remains relatively clean. A particularly revealing observation comes from a nanopillar that was split open during cleaving (Figure 3e,f). Beneath the layer of densely deposited nanoparticles on its surface, concealed nanostructures resembling LIPSS with periods on the order of hundreds of nanometers are exposed. The development of the deep tapered micro-holes and elevated rims is elucidated by a model illustrated in Figure 3g. The morphological evolution is governed by distinct mechanisms in the central and peripheral regions, dictated by the local energy distribution of the Bessel beam. In the central region, which comprises the main lobe and adjacent high-energy side lobes, intense energy deposition induces both direct material removal and a steep thermal gradient, the latter driving a robust Marangoni flow [41,42]. This flow radially propels molten material from the hotter center toward the cooler periphery, where it resolidifies, forming the characteristic elevated rims. This subtractive and transformative process concurrently clears the optical path, facilitating the beam’s downward propagation. This propagation is sustained by diffraction and interference within the beam itself, combined with reflections from the cavity sidewalls. As a result, the beam re-concentrates intense energy at the bottom of the cavity, generating new hot spots that drive further ablation and create a self-reinforcing cycle (Figure 3g). This propagation and intensification mechanism is directly supported by numerical simulations of the electric field distribution within representative cavities (Figure 3h). The simulations reveal that, despite a discrete field pattern arising from internal reflections and interference, significant energy is consistently concentrated near the bottom region even in deep (e.g., 27 μm) cavities. This confirms that the Bessel beam’s self-reconstructing property, aided by sidewall reflections, enables effective energy delivery to the bottom of high-aspect-ratio structures, thereby validating the proposed dynamic cycle. In contrast, within the outer-side lobes where energy is moderate, the conditions are sufficient only for the formation of laser-induced periodic surface structures (LIPSS), resulting in the characteristic nanoscale gratings observed in these annular regions. The formation of the adherent nanoscale coating originates from the re-deposition of the laser-induced plasma plume and ejected material. This deposition process is predominantly fueled by the moderate energy density of the outer-side lobes, as well as the lateral incident light preceding its transformation into the Bessel beam’s interference pattern. This peripheral energy level is sufficiently high to melt the surface and sustain the agglomeration of re-deposited species into nanoparticles, yet sufficiently mild to prevent the intense ablation and fluid dynamics that dominate the central region [14,17]. The presence of these nanostructures is further corroborated by the ripples observed in Figure 2 and within the split nanopillar in Figure 3e,f, which suggest a formative role of the side lobes in the deposition process.

**Figure 4:**
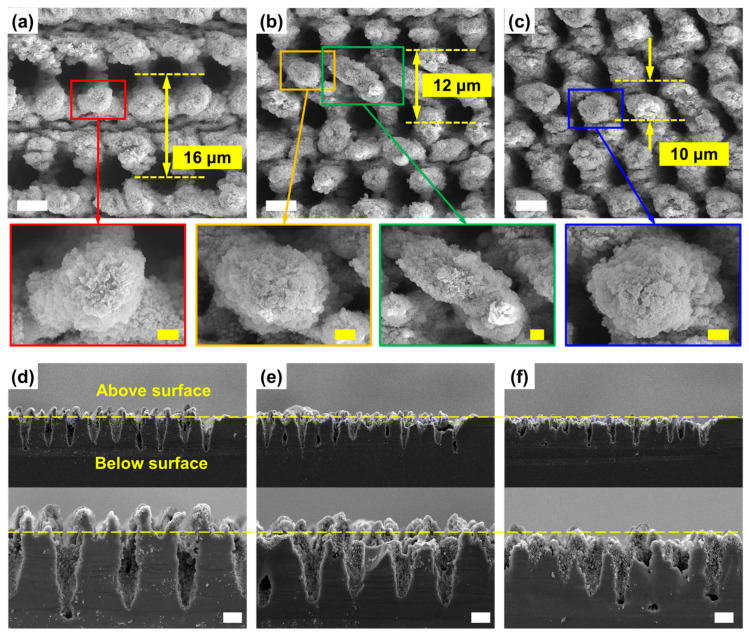
Micro–nano hierarchical structure morphologies with areal scanning. (**a**–**c**) Top-view SEM images with line adjacencies of (**a**) 16 μm, (**b**) 12 μm, and (**c**) 10 μm. (**d**–**f**) Side view SEM images with line adjacencies respectively corresponding to (**a**–**c**). Scale bar: 5 μm in the main figures (white ones) and 1 μm in the inserted picture of (**a**–**c**) (yellow ones).

Figure 4 presents the morphologies of the micro–nano hierarchical structure fabricated via areal scanning with different line intervals. As shown in Figure 4a–c, the surface coverage and structural continuity are highly dependent on this parameter. At a large line interval of 16 μm (Figure 4a), the micro–nano structures generally cover the surface, but distinct micron-scale gaps remain between adjacent scan lines. Within these gaps, clear ablation traces from the side lobes of the Bessel beam are evident. When the line interval is reduced to 12 μm (Figure 4b), the gaps between lines disappear. At this spacing, the micropillars from neighboring rows begin to interact, showing signs of partial merging or coalescence. The magnified images within the yellow and green frames exemplify isolated and merged pillars, respectively. A further reduction in the interval to 10 μm (Figure 4c) leads to the extensive merging of pillars from adjacent rows, forming a more continuous network. The high-magnification SEM images confirm that, regardless of their merging state, all pillars are uniformly coated with a dense layer of nanostructures. The corresponding side-view images (Figure 4d–f) reveal further insights into the structural evolution. As the line interval decreases from 16 μm to 10 μm, the height of the micropillars protruding above the original surface progressively diminishes. Notably, at the 10 μm interval, the distinct above-surface pillar morphology vanishes entirely. Correspondingly, the depth of the underlying tapered micro-holes also decreases with the reduction in line spacing. The observed phenomena can be explained by considering the formation mechanism of the Bessel beam itself. A Bessel beam is formed by the constructive interference of conical waves incident from annular regions. The integrity and stable propagation of the beam rely on contributions from all these azimuthal directions. Consequently, blocking any portion of this incident light perturbs the beam’s profile. During areal scanning with a reduced line interval, the pre-fabricated micropillars from previous scans act as physical obstructions, shadowing and disrupting the incident light required to form the ideal Bessel beam for subsequent drilling. This perturbation directly degrades the beam’s ability to maintain its intense central core, leading to the observed reduction in micro-hole depth. Simultaneously, these same micropillars are themselves subjected to laser ablation by the incoming beam, resulting in their progressive truncation and eventual eradication at the smallest line interval.

## 4. Conclusions

In conclusion, we have demonstrated a rapid drilling-on-the-fly strategy for silicon using an emission-programmed, high-repetition-rate femtosecond Bessel beam. This approach enables the single-step fabrication of true 3D hierarchical micro–nano structures, integrating tapered micro-holes, elevated micropillars, and a dense coating of nanostructures. By spatially coupling distinct material modification modes (subtractive, transformative, and additive processes) within one beam profile, it accomplishes structural complexities that are challenging to achieve with conventional techniques. Through a quasi-reconstructed analysis of the structural evolution via SEM, the formation mechanism was deciphered as the integration of subtractive, transformative, and additive processes: the central main lobe and adjacent high-energy side lobes drive a dynamic process involving subtractive ablation and transformative thermo-fluidic redistribution, while the outer-side lobes with moderate energy facilitate the additive generation of nanostructures. Furthermore, we show that the resulting depth of the micro-holes and the height of the micropillars can be precisely tailored by controlling the line interval during areal scanning, allowing for programmable geometry in the fabricated arrays. Compared with conventional continuous-output laser processing, our emission-programming strategy provides superior temporal control at the level of individual pulse trains—a precision unattainable through mere adjustment of pulse energy or scan speed. This capability holds the potential to enable novel fabrication modalities, such as single-step creation of surfaces with spatially graded properties by locally varying the temporal sequence of pulse trains. However, this fine temporal control inherently reduces the net average power compared to continuous operation at the same repetition rate, establishing a practical trade-off between control fidelity and processing throughput. As a result, the current processing rate of 500 holes/s at 4000 μm/s is primarily constrained by the available pulse energy at the laser’s intrinsic high repetition rate (fp). Looking forward, employing laser sources that deliver higher pulse energies at elevated repetition rates would allow the same programmed energy per hole to be delivered at proportionally higher scanning speeds (e.g., ~10,000 μm/s) and pulse-train frequencies. Thus, this method establishes a scalable framework in which advancements in laser technology directly translate to enhanced throughput without altering the underlying temporal control scheme.

In perspective, the elucidated light-matter interaction and formation mechanism of the micro–nano structures suggest the broad applicability of both the resulting structures and the processing method. From a material standpoint, the proposed strategy and the underlying physical mechanisms are rooted in fundamental material responses to ultrafast laser irradiation, such as strong near-infrared absorption and consequent melt-flow dynamics. Therefore, they are inherently transferable to a broad range of non-transparent materials, including other semiconductors (e.g., Ge, GaAs) and metals (e.g., Ti, stainless steel). This universality positions the resulting hierarchical structures as promising candidates for applications in photodetectors, biocompatible surfaces, and drag-reduction coatings. Specifically, the ability to tune micropillar height via the scanning line interval provides a direct pathway for in-depth investigation and precise control of drag-reduction properties. From a processing perspective, tailoring the incident energy and the profile of the Bessel beam offers a potential route to control key parameters of the tapered micro-holes, such as depth and cone angle, for diverse applications. Furthermore, other beam profiles possessing a similar main lobe–side lobe structure and relying on a diffraction-interference balance could yield analogous structuring effects. In summary, this work provides a fresh viewpoint on complex laser-matter interactions and is anticipated to stimulate further application of laser-induced micro–nano hierarchical structures in related fields.

## Data Availability

Data is available from the authors upon request.
